# Cost-Efficient and Multi-Functional Secure Aggregation in Large Scale Distributed Application

**DOI:** 10.1371/journal.pone.0159605

**Published:** 2016-08-23

**Authors:** Ping Zhang, Wenjun Li, Hua Sun

**Affiliations:** 1 School of Electronics and Information Engineering, Hunan University of Science and Engineering, Hunan, P.R. China; 2 Hunan Provincial Key Laboratory of Network Investigational Technology, Hunan Police Academy, Hunan, P.R.China; 3 Hunan Provincial Key Laboratory of Intelligent Processing of Big Data on Transportation, Changsha University of Science and Technology, Hunan, P.R. China; 4 School of Information Engineering, Changsha Medical University, Hunan, P.R. China; Universita degli Studi di Catania, ITALY

## Abstract

Secure aggregation is an essential component of modern distributed applications and data mining platforms. Aggregated statistical results are typically adopted in constructing a data cube for data analysis at multiple abstraction levels in data warehouse platforms. Generating different types of statistical results efficiently at the same time (or referred to as enabling multi-functional support) is a fundamental requirement in practice. However, most of the existing schemes support a very limited number of statistics. Securely obtaining typical statistical results simultaneously in the distribution system, without recovering the original data, is still an open problem. In this paper, we present SEDAR, which is a SEcure Data Aggregation scheme under the Range segmentation model. Range segmentation model is proposed to reduce the communication cost by capturing the data characteristics, and different range uses different aggregation strategy. For raw data in the dominant range, SEDAR encodes them into well defined vectors to provide value-preservation and order-preservation, and thus provides the basis for multi-functional aggregation. A homomorphic encryption scheme is used to achieve data privacy. We also present two enhanced versions. The first one is a Random based SEDAR (REDAR), and the second is a Compression based SEDAR (CEDAR). Both of them can significantly reduce communication cost with the trade-off lower security and lower accuracy, respectively. Experimental evaluations, based on six different scenes of real data, show that all of them have an excellent performance on cost and accuracy.

## Introduction

Enormous amounts of rich diverse information are constantly generated in modern large distributed systems, which are also called big data. Such large-scale big data sources create exciting opportunities for service quality monitoring, novelty discovery, or attack detection, etc. However, directly transmitting them to a single node and processing using centralized algorithms are difficult. Distributed aggregation is an efficient way to minimize consumption of energy and bandwidth.

Typical distributed application scenarios can be easy to found big internet firms, such as Google and Bing. Click log data of these service providers is distributed on thousands of servers around the world, which is usually up to megabytes per minute. In these distributed big data scenarios, 90% of regular analytics jobs issue queries against different type of aggregated values, instead of requiring the raw log data records. To efficient generate these aggregated results, performance metrics is generated locally from log data, and then a distributed aggregation can be adopted [1].

As another example, consider the WSNs application scenarios. In these applications, nodes are often equipped with a battery as the energy unit, which means the energy capacities are limited. Meanwhile, such WSNs are envisioned to be spread out over a large geographical area, and the total number of the nodes is huge, so the battery change is impossible. How to save the overall energy resources and extend the lifetime of the networks is essential. Distributed aggregation is also a popular research topic in this area [2].

Enabling multi-functional support is a fundamental requirement in practice. Here, multi-functional support means to provide as many statistical results as possible. Typical aggregation functions include count, summation, mean, median, maximum, minimum, variance, mode, etc. These statistical results are typically adopted in constructing a data cube for data analysis at multiple abstraction levels in data warehouse platforms [3], In order to improve the performance of data mining, it is a basic requirement to keep data feature (i.e. statistics) as much as possible in data cubes, which means that enabling multi-functional support is necessary for corresponding distributed aggregation schemes. System wide properties generated from data aggregation, can also be used as input parameters for other distributed applications and algorithms, or utilized for decision making directly. For example, setting the fan-out of gossip protocol [4] in peer-to-peer applications, or achieving load balancing in content delivery networks [5] need aggregation results as their parameters or inputs.

Serval distributed aggregation schemes [6–8] have been put forward. Security is a basic requirement for most applications. Serval secure distributed aggregation schemes [2, 9–13] have likewise existed. However, most of them can only achieve a very limited type of statistics, and even combine several existed schemes still can’t respond to the request. In fact, efficient obtaining global-related statistical results, such as median and mode, in a distributed manner, even without considering security problem, is still a challenge [3].

In RCDA [14], a homomorphic encryption algorithm is used to provide end-to-end confidentiality, and simple concatenation all sensing data without any information compression method to enable recoverability of all sensing data. Although the scheme can achieve arbitrary method support, the communication cost is too heavy to be applied to large scale networks. Based on RCDA, EERCDA [13] uses a differential data transfer method to reduce the communication cost, in which the difference data rather than raw data from the sensor node are transmitted to the cluster head. However, the total transmission overhead still too heavy for most time.

To the best of our knowledge, securely obtaining typical statistical results simultaneously in the distribution system, without recovering the original data, is still an open problem.

In this paper, we study the problem of *multi-functional secure distributed aggregation*, in which all the aggregation functions mentioned above can be obtained securely in a single aggregation query. We also propose three complementary schemes to work around this problem. We first present SEDAR, which is a SEcure Data Aggregation scheme under the Range segmentation model, and then proposed two enhanced version REDAR (Random based SEDAR) and CEDAR (Compression based SEDAR).

To reduce the communication cost by capturing the data characteristics, a range segmentation model is adopted in proposed schemes, and different range uses different aggregation strategy. Raw data in dominant range are encoded into well defined vectors at each node to preserve both the order-related and the value-related information during distributed aggregation, and thus different types of statistics can be obtained simultaneously recovering the original data. The vectors are encrypted by a homomorphic scheme, and encrypted vectors are aggregated directly in cipher domain at an intermediate node, so concealment is also achieved. Raw data in other range will be encrypted by traditional asymmetric encryption schemes, and transmitted without in-network aggregation.

The major contributions of this paper are summarised as follows.

- We propose a novel and practical scheme, called SEDAR, in which all common statistical results can be securely and efficiently obtained without recovering the original data.
- We also present two enhanced versions, namely REDAR and CEDAR, to further reduce the communication cost with the trade-off lower security and lower accuracy, respectively.
- We implement these three schemes and extensively evaluate their performance. Evaluation results, based on six different scenes of real data, show that all of them have an excellent performance on cost and accuracy.

The remaining parts of this paper are structured as follows. Section 2 describes terminologies, and additional background knowledge. Section 3, 4 and 5 introduce SEDAR, REDAR and CEDAR. Section 6 and 7 is performance analysis and evaluation results. Section 8 briefly examines the related work. Section 9 provides a summary.

## Preliminaries

In this section, we first give a range segmentation model and a network model, and then present problem definition. We also introduce a homomorphic encryption scheme.

### Range Segmentation Model

**Fig 1:**
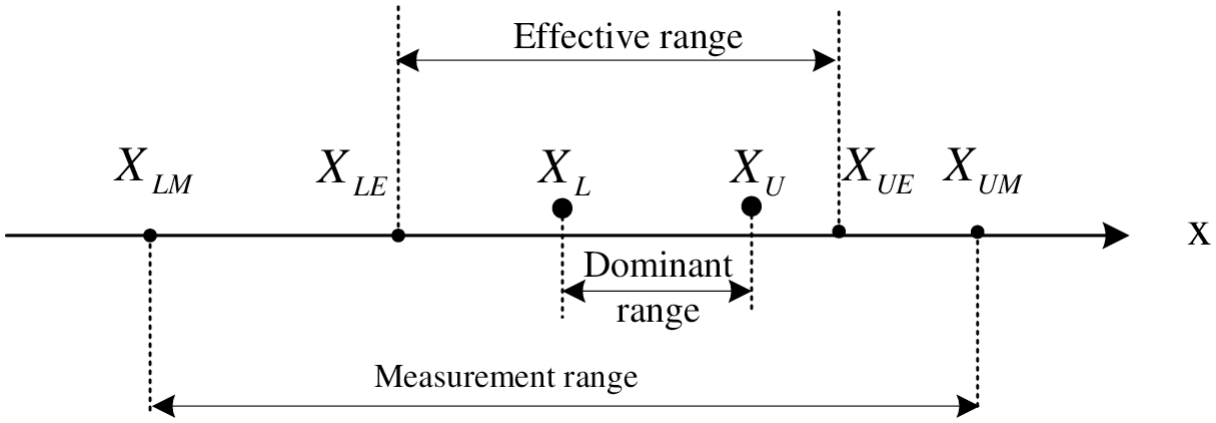
Range Segmentation Model.

An illustration of range segmentation model is given in Fig 1, terminologies used in this model are defined as follows.

**Definition 1** *(R*_*m*_, *measurement range) Measurement ranges are those over which the measurement instruments are calibrated. Convincing and reliable results of a given instrument will only appear in its measurement range, i.e.,* *R*_*m*_ = [*X*_*LM*_, *X*_*UM*_], *s*.*t*. *R*_*m*_ ⊆ *R*

**Definition 2** *(R*_*e*_, *effective range, operation range, valid range) Effective range is the set of allowed values for a variable in a concrete application. It’s a subset of* *R*_*m*_, *i.e.,* *R*_*e*_ = [*X*_*LE*_, *X*_*UE*_], *s*.*t*. *R*_*e*_ ⊆ *R*_*m*_

**Definition 3** *(R*_*d*_, *dominant range, dominant area, advantaged region, main range) Dominant range is a subset of R_*e*_, whose probability is significantly greater than other’s, i.e.,* *R*_*d*_ = [*X*_*L*_, *X*_*U*_] *s*.*t*. *R*_*d*_ ⊆ *R*_*e*_ && *P*(*R*_*e*_) − *P*(*R*_*d*_) ≪ 1

**Definition 4** *(R*_*b*_, *border region, boundary region, margin area) Border region is the set of allowed values that outside the dominant range. It’s a subset of* *R*_*e*_, *i.e.,* $R_{b}=[X_{L}{}_{E},X_{U}{}_{E}]={\bar{R}}_{d}R_{e}\;s.t.R_{b}\cap R_{d}=\emptyset \;\&\&\;R_{b}\cup R_{d}=R_{e}$

### Network Model

The network is modeled as a connected graph *G* = (*V*, *E*), with |*V*| vertices and |*E*| links. Each vertex represents a network node and each link represents a communication channel. Node is a logical concept. For example, in global scale distribution systems, each data center can be regarded as a node.

The sink node *S* ∈ *V*, which has a powerful computing and storage capacity, is a trusted node. *S* is also known as query server. The remainder nodes *C* ⊂ *V* are either reliable or unreliable, each node only has one parent node. |*C*| = *N*. **X** = {*x*_1_, *x*_2_, …, *x*_*N*_} is raw data generated at these nodes. A set of nodes *A* are selected as aggregator nodes, *A* ⊂ *C*. The aggregator nodes are also performed as cluster heads, the others nodes ($C\bar{A}$) are cluster members. Each cluster member join an appropriate cluster according certain criterion, such as signal strength in wireless network and delay in wired network.

### Problem Definition

**Definition 5** *(Data aggregation) Given a dataset* *X* = {*x*_1_, *x*_2_, …, *x*_*N*_}, *a aggregation function set* *F* = {*f*, *h*, …}, *and a aggregation result set* *Y* = {*y*_1_}, *y*_2_, …, *a data aggregation is defined as* *Y* = *F*(*X*), *s*.*t*. |*Y*| ≪ |*X*|.

**Definition 6** *(Distributed Aggregation) Given a network G, X is the raw data generated at each node, divide X into several subsets* {*X*_1_, *X*_2_, *X*_*M*_}, s.t. *M* < *N*, *X*_1_ ∪ *X*_2_… ∪*X*_*M*_ = *X*, *X*_1_ ∩ *X*_2_… ∩*X*_*M*_ = *ϕ*. *An in-network data aggregation is defined as* *y* = *F*(*X*) = f (h(*X*_1_),…, h(*X*_*M*_)). *Each subset can be further divided, and this definition is still satisfied*.

Data aggregation of each subset is accomplished at aggregator nodes, and the final data aggregation is executed at the query server.

**Definition 7** *(MFSDA, Multifunction Secure Distributed Aggregation) An MFSDA is a distributed aggregation which can provide both privacy confidentiality and multi-functional supporting. Multi-functional means that several statistical results can obtain efficiently in the same query, and results include at least count, summation, average, median, maximum, minimum, variance and standard deviation.*

### Homomorphic Encryption Scheme

The traditional encryption technology is not suitable for secure distributed aggregation. It only provides concealment but do not support cipher text operations, so the intermediate aggregators will have to decrypt the received data before aggregation. And then the aggregated results need be re-encrypted before sending. Frequent encryption and decryption in intermediate nodes will increase the computing cost and the energy consumption. The key management is also difficult. Each intermediate node has to maintain the private key for decryption, which will increase the risk of leaks.

To reduce the computing cost and enhance the security, a homomorphic encryption scheme is used in the proposed schemes. It is derived from homomorphism in the abstract algebra. By using homomorphism, operations in one algebraic system (plaintext) can be mapped into operation in another algebraic system (cipher text), which means data aggregation can perform on cipher text directly, and only the sink node needs to store the private for decryption. Homomorphic encryption technology includes partially homomorphic and fully homomorphic. In theory, based on the fully homomorphic, all these statistics can be easily computed. However, the fully homomorphic encryption, while revolutionary, is not really practical. Practitioners rely therefore on already existing partially homomorphic encryption, which is constructed from traditional encryption schemes and has been widely used in multiparty computation, electronic voting, non-interactive verifiable secret sharing, e-auction, and others [15, 16].

Homomorphic encryption used in proposed schemes is a partially homomorphic which can only allow homomorphic computation of only one operation (i.e., addition). To further reduce key size with high security and benefit us with the computation cost, an ElGamal Encryption Scheme (EC-EG) [2, 14], which is an elliptic curve based encryption, will be used here. EC-EG is also an asymmetric homomorphic encryption scheme, so the key management is easy. It consists of four parts: Setup, KeyGen, Encryption (HEnc) and Decryption (HDec). Its ciphertext is an elliptic curve over a finite field, and ⊕ is the point addition on elliptic curves.

**Theorem 1 (Additive Homomorphic Encryption)** *EC-EG is an additive homomorphic encryption scheme, namely the addition in plaintext is equivalent to point addition in cipher domain, i.e.,* *m*_1_ + *m*_2_ = *HDec*(*HEnc*(*m*_1_) ⊕ *HEnc*(*m*_2_))

## SEDAR

In this section, we introduce SEDAR to solve the *multi functional secure distribution aggregation* problem. We first give a brief overview of SEDAR. Then, a detail version is presented. Finally, a concrete example is given.

### Overview

In the proposed scheme, aggregation is performed in cipher domain. Both the sub-aggregation and the final aggregation results are encrypted vectors, which can be decrypted using the private key owned by the server. All statistics are calculated directly from the final aggregated vector at the server, which makes thing much easier.

**Fig 2:**
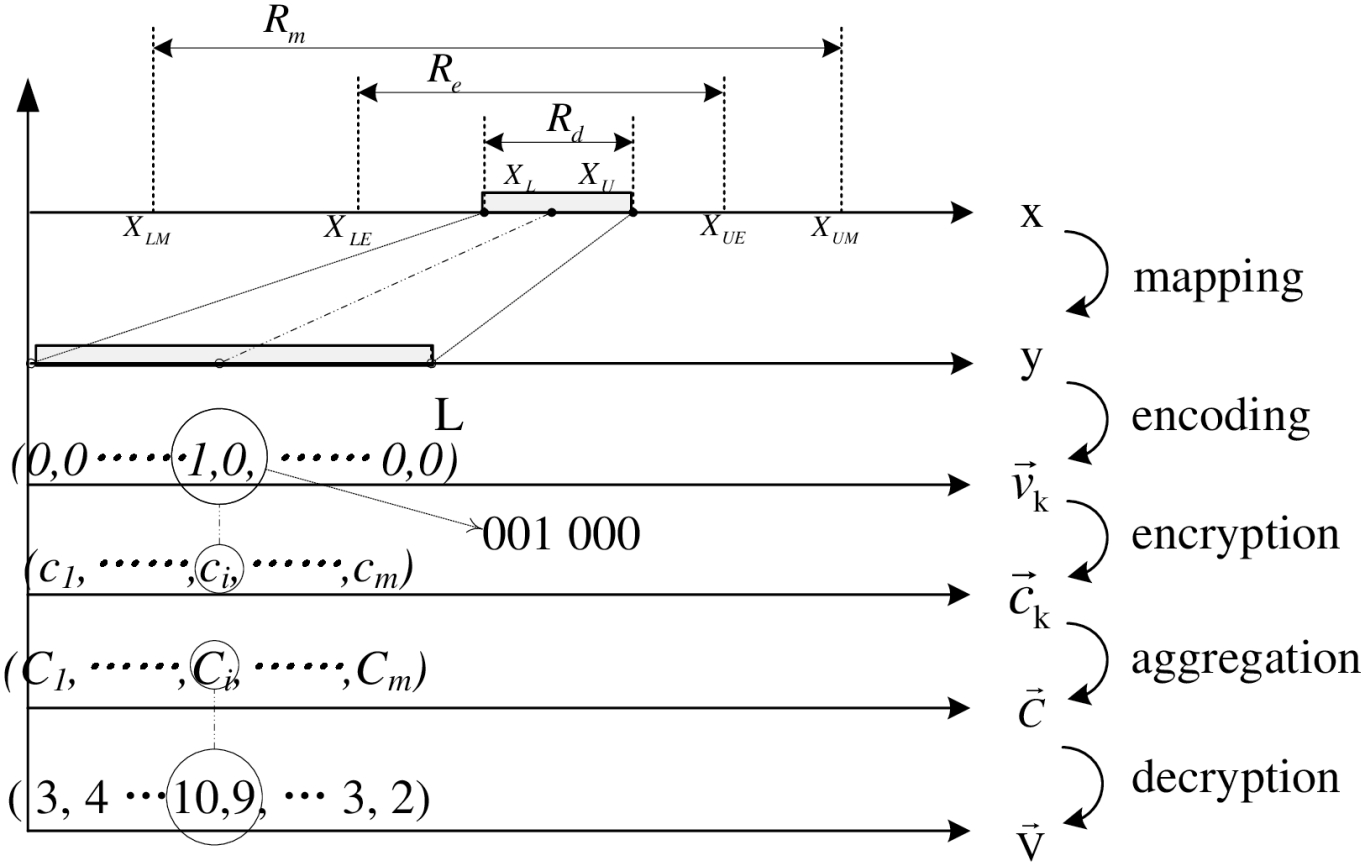
SEDAR.

As shown in Fig 2, there are five steps in the proposed scheme: mapping, encoding, encryption, aggregation, and decryption. The first three are executed on each node independently, while the last two are executed at aggregation nodes and the server respectively. Mapping and encoding are used to enable multi-function supporting, while the other three are used to achieve data confidentiality.

There are also five kinds of data corresponding to each step: raw data *x*, mapped data *y*, encoded data $\vec{v}$, encrypted data $\vec{c}$, aggregation of encrypted data $\vec{C}$, and aggregation of encoded data $\vec{V}$.

*Raw data* *x*_*k*_ is the original data gathered at node *k*, which is belong to a subset of real domain. This real domain is split into several partition based on the range segmentation model, and different strategies will choice for different range.

The lower bound of *R*_*d*_ is defined as $X_{L}=max(X_{L}{}_{E},\hat{\mu }-\beta \hat{\delta })$, and the upper one is $X_{U}=min(X_{U}{}_{E},\hat{\mu }+\beta \hat{\delta })$. $\vec{\mu }$ and $\vec{\delta }$ are mean and standard deviation estimated using historical or empirical data. *β* is a factor. *β* ∈ [1.8, 3], and *β* = 2 satisfies most application. The bounds of *R*_*e*_ and *R*_*m*_ are determined by the application itself.

Effective data in the dominant range (*x*_*k*_ ∈ *R*_*d*_) will be transformed into *mapped data* *y*_*k*_ using the *mapping* function. *y*_*k*_ is belong to a subset of the natural numbers, i.e. *y*_*k*_ ∈ (0, *L*], where $L=\;\left\lceil \frac{X_{U}-X_{L}}{a}\right\rceil$ and *a* is the accuracy requirement of *x*_*k*_. The conversion between *x*_*k*_ and *y*_*k*_ is achieve by the *mapping* function and its inverse, i.e. *f*_*m*_ and $f_{m}^{-1}$. To achieve value-preserved and order-preserved during this conversion, *f*_*m*_ and $f_{m}^{-1}$ should be monotonic functions. The mapping function can be a linear one or a nonlinear one, which can be either a piecewise function or a non piecewise function. For example, a linear *mapping* function and its inverse are like $y_{k}=f_{m}(x_{k})=\;\frac{x_{k}-X_{L}{}_{B}}{a}$, and $x_{k}=f_{m}^{-1}(y_{k})=a\times y_{k}+X_{L}{}_{B}$ .

Effective data outside the dominant range (*x*_*k*_ ∈ *R*_*b*_) will be encrypted by traditional asymmetric encryption scheme, and transmitted without in-network aggregation. As *P*(*R*_*e*_) − *P*(*R*_*d*_) ≪ 1, serial transmission of these data will not increase the transmission overhead significantly. Abnormal data that outside *R*_*e*_ are also reported to the server without aggregation.

Encoded data ${\vec{v}}_{k}$ is a vector, ${\vec{v}}_{k}\in \{0,1{\}}^{L}$, where *L* is the number of elements. The (*y*_*k*_)*th* element of ${\vec{v}}_{k}$ is 1, and all other elements is 0. The conversion between *y*_*k*_ and ${\vec{v}}_{k}$ is achieve by the *encoding* function and its inverse, i.e., ${\vec{v}}_{k}=f_{e}(y_{k})$, and $y_{k}=f_{e}^{-1}({\vec{v}}_{k}).$ For example, the encoding function can be programmed as two instructions, i.e. ${\vec{v}}_{k}=zeros(1,L)$ and ${\vec{v}}_{k}(y_{k})=1$, while its inverse function can be achieved by $y_{k}=find({\vec{v}}_{k}>0)$.

Each node k encrypts its ${\vec{v}}_{k}$ into ${\vec{c}}_{k}$ using the homomorphic encryption scheme, and sent ${\vec{c}}_{k}$ to its parent. As all encrypted data ${\vec{c}}_{k}$ are generated with the same public key, we can aggregation it directly in ciphertext $\vec{C}=⊕{\vec{c}}_{k}$. According homomorphism, the final aggregation result $\vec{V}$ can be obtain by decrypting $\vec{C}$. Then the typical statistical results can be obtained from $\vec{V}$.

### Detail of SEDAR

*SEDAR* consists of three procedures: *Setup*, *Operations on Clients*, and *Operations on Server*.

#### Setup

The *Setup* procedure performs network initialization, encryption initialization, and parameters distribution.

Boundary definitions (i.e., *R*_*e*_, *R*_*d*_, and *R*_*b*_) and accuracy requirement (i.e. *a*) are distributed into each node, include clients and server. *Itm*_*size*_, *B*_*msize*_, *B*_*csize*_, and *B*_*num*_ mentioned above are also public information.

There are two encryption schemes used in this paper: a traditional one and a homomorphic one. Both of them are public key cryptogram schemes.

The traditional encryption scheme is used for *R*_*b*_, where the key pair is {*KPriS*, *KPubS*}, the encryption function is *C* = *Enc*(*msg*, *KPubS*), and the decryption function is *msg* = *Dec*(*C*, *KPriS*).

The homomorphic encryption scheme is used for *R*_*d*_, where the key pair is {*KPriSH*, *KPubSH*}, the encryption function is *C* = *HEnc*(*msg*, *KPubSH*), and the decryption function is *msg* = *HDec*(*C*, *KPriSH*).

Both *KPubS* and *KPubSH* are public information, while private keys (i.e. *KPriS* and *KPriSH*) must be keep in privacy only by the server.

#### Operations on Clients

It consists of three parts: *local data processing*, *received data processing* and *data transmission*.

As shown in algorithm 1, in *local data processing*, each node gathers the raw data *x*_*i*_, and process it according the range definition. The traditional encryption scheme will be used for the data belong to *R*_*b*_, the encrypted data will add into *bSet*_*i*_. For data in *R*_*d*_, *mapping* *f*_*m*_ and *encoding* *f*_*e*_ function will be used before the homomorphic encryption, the encrypted data will add into *hSet*_*i*_. For the data outside the valid range *R*_*e*_, the node ID will be added into the alarm set *aSet*_*i*_ after being encrypted.

**Algorithm 1** Operations on Client (Part I)

1: **procedure** local data processing

2:  **if** (*x*_*i*_ ∈ *R*_*b*_) **then**

3:   *C*_*i*_ ← *Enc*(*x*_*i*_, *KPubS*)

4:   *bSet*_*i*_ ← {*C*_*i*_}

5:  **else if** (*x*_*i*_ ∈ *R*_*d*_) **then**

6:   *y*_*i*_ ← *f*_*m*_(*x*_*i*_)                      ⊳ Mapping

7:   ${\vec{v}}_{i}\leftarrow f_{e}(y_{i}),\;\;\;$ s.t. ${\vec{v}}_{i}\in \{0,1{\}}^{L}$.          ⊳ Encoding

8:   *C*_*hi*_ ← *nul*

9:   **for** *j* ← 0 to *B*_*num*_ − 1 **do**

10:    $start\leftarrow max(1,(|{\vec{v}}_{i}|(-(j+1)B_{msize}+1)$

11:    $end\leftarrow |{\vec{v}}_{i}|-jB_{msize}$

12:    $m\leftarrow {\vec{v}}_{i}[start,end]$

13:    *c* ← *HEnc*(*m*, *KeyPubSH*)

14:    *C*_*hi*_ ← [*c*, *C*_*hi*_]

15:   **end for**

16:   *hSet*_*i*_ ← {*C*_*hi*_}

17:  **else**

18:   *CID*_*i*_ ← *Enc*(*ID*_*i*_, *KPubS*)

19:   *aSet*_*i*_ ← {*CID*_*i*_}

20:  **end if**

21: **end procedure**

As shown in algorithm 2, the *received data processing* only exists in cluster header (i.e., CHs). Each CH use it to deal with packets received from its children (i.e., CMs). Items in each packet will be classified into three sets, i.e., *bSet*_*i*_, *hSet*_*i*_, and *aSet*_*i*_. All items of *hSet*_*i*_ will be aggregated directly in cipher domain, and the aggregation result is *Ch*_*i*_.

In the *data transmission*, all processing results *Ch*_*i*_, *bSet*_*i*_, and *aSet*_*i*_ are send to its parent.

Each element in ${\vec{v}}_{k}$ (i.e., *v*_*k*_(*i*)) or $\vec{V}$ is allocated the same size (denote as, *Itm*_*size*_ or |*v*_*k*_(*i*)|), it is influenced by *N* and the distribution of *x*. $Itm_{size}\in (\lceil log\frac{N}{L}\rceil ,\lceil logN\rceil )$.

The maximum size (denote as *P*_*size*_) that the homomorphic encryption function can operate each time, is always large than *Itm*_*size*_. To reduce the total ciphertext size and the computation cost, several adjacent vector elements can be encrypted at the same time. For example, in Fig 2, each element is allocated three bits, and every two elements are encrypted with each other.

The maximum number of vector elements that can be encrypted by the encryption function is $\left\lfloor \frac{P_{size}}{Itm_{size}}\right\rfloor$, and the actual size of plaintext for the encryption function is $B_{msize}=\lfloor \frac{P_{size}}{Itm_{size}}\rfloor Itm_{size}$. The corresponding ciphertext size is denoted as *B*_*csize*_. When $\left|{\vec{v}}_{k}\right|$ is much larger than *B*_*msize*_, the homomorphic encryption function need to repeat $B_{num}=\left\lceil \frac{\left|{\vec{v}}_{k}\right|}{B_{msize}}\right\rceil$ times to finish the encryption for ${\vec{v}}_{k}$. The decryption function also needs to repeat *B*_*num*_ times.

**Algorithm 2** Operations on Client (Part II)

1: **procedure** received data processing

2:  **if** current node is a CH **then**

3:   **for all** received *Packet*_*k*_ **do**

4:    extract {*C*_*hk*_, *bSet*_*k*_, *aSet*_*k*_} from *Packet*_*k*_

5:    *bSet*_*i*_ ← *bSet*_*i*_ ∪ *bSet*_*k*_

6:    *aSet*_*i*_ ← *aSet*_*i*_ ∪ *aSet*_*k*_

7:    *hSet*_*i*_ ← *hSet*_*i*_ ∪ {*C*_*hk*_}

8:   **end for**

9:   **for** *j* ← 1 to *B*_*num*_ **do**

10:    *ct*_1_ is initialized as the *infinity* point of *E*

11:    **for all** *C*_*hk*_ ∈ *hSet* **do**

12:     *ct*_2_ ← *C*_*hk*_ [(*j* − 1)*B*_*csize*_ + 1, *jB*_*csize*_]

13:     *ct*_1_ ← *ct*_1_ ⊕ *ct*_2_

14:    **end for**

15:    *C*_*hi*_ [(*j* − 1)*B*_*csize*_ + 1, *jB*_*csize*_]←*ct*_1_

16:   **end for**

17:  **end if**

18: **end procedure**

#### Operations on Sever

Operations on *server* consist of five parts: *data receiving and retrieving*, *boundary range data processing*, *alarm data processing*, *dominant range data processing*, and *obtain statistical results*. The first three are contained in algorithm 3, while the others are contained in algorithm 4 and 5.

**Algorithm 3** Operations on Server (Part I)

1: **procedure** operations on server

2:  **for all** received *Packet*_*k*_ **do**                      ⊳ data retrieving

3:   extract {*C*_*hk*_, *bSet*_*k*_, *aSet*_*k*_} from *Packet*_*k*_

4:   *bSet* ← ⋃*bSet*_*k*_

5:   *aSet* ← ⋃*aSet*_*k*_

6:   *hSet* ← ⋃*C*_*hk*_

7:  **end for**

8:  *mSet* ← {}                      ⊳ boundary range data processing

9:  **for all** *C*_*i*_ ∈ *bSet* **do**

10:   *m*_*i*_ ← *Dec*(*KPriS*, *C*_*i*_)

11:   *mSet* ← ⋃ {*m*_*i*_}

12:  **end for**

13:  **for all** *cid* ∈ *aSet* **do**                     ⊳ alarm data processing

14:   *ID*_*i*_ ← *Dec*(*KPriS*, *cid*)

15:   treat *ID*_*i*_ as a potential abnormal node

16:  **end for**

17: **end procedure**

In the *data receiving and retrieving*, the server receives all packets from its children. All its children are CH, and the packets like {*C*_*hi*_, *bSet*_*i*_, *aSet*_*i*_}. Items in each packet will be classified into three sets, i.e. *bSet*, *aSet* and *hSet*.

Items in *bSet* are boundary range data, all of them are encrypted in traditional scheme. The decrypted data are added into *mSet*.

Items in *aSet* are IDs of clients which data is out of boundary range. Those nodes are treated as potential abnormal node, and may need further analysis.

Items in *hSet* are dominant range data, all of them are homomorphic encrypted data, so all items in this set can be aggregated directly in cipher domain. After decrypting the aggregated encrypted data using *KPriSH*, we get an aggregation result of data in dominant range, in a vector form, i.e., $\vec{V}=\{n_{1},n_{2},\ldots ,n_{L}\}$.

**Algorithm 4** Operations on Server (Part II)

1: **procedure** Operations on Server

                        ⊳ dominant range data processing

2:  **for** *j* ← 1 to *B*_*num*_ **do**

3:   *ct*_1_ is initialized as the *infinity* point of *E*

4:   **for all** *C*_*i*_ ∈ *hSet* **do**

5:    *ct*_2_ ← *C*_*i*_ [(*j* − 1)*B*_*csize*_ + 1, *jB*_*csize*_]

6:    *ct*_1_ ← *ct*_1_ ⊕ *ct*_2_

7:   **end for**

8:   *tmp* ← *HDec*(*t*_1_, *keyPriS*)

9:   $\vec{M}\leftarrow [\vec{M},tmp]$

10:  **end for**

11:  $\vec{V}\leftarrow nul$

12:  $B_{size}\leftarrow \frac{\left|M\right|}{L}$

13:  **for** *j* ← 1 to *L* **do**

14:   *n*_*j*_ ← *M* [|*M*| −*jItm*_*size*_ + 1, |*M*| −(*j* − 1)*Itm*_*size*_]

15:   $\vec{V}\leftarrow [n_{j},\vec{V}]$

16:  **end for**

17: **end procedure**

Finally, each statistical results can be obtained directly from $\vec{V}$ and *mSet* by algorithm 5.

### Property of SEDAR

#### Multi-function

On the one hand, the value-related information needs to be preserved in the transformation for the summation based statistics. *y*_*k*_ can be recovered from $\vec{V}(i)$, and *x*_*k*_ can be recovered from *y*_*k*_. $\vec{V}(i)$ itself represents how many $x_{k}=f_{m}^{-1}(i)$ in the raw data. So the value-related information is maintained in the $\vec{V}$.

On the other hand, the order-related information needs to be preserved in the transformation for the comparison based statistics. Assuming that $\vec{V}(i)\neq 0$, $\vec{V}(j)\neq 0$, and *i* > *j*. We recover the raw data as $x_{i}=f_{m}^{-1}(i)$ and $x_{j}=f_{m}^{-1}(j)$, and then we can use the monotonicity of *f*_*m*_ to judge which one is larger. So order-related information is maintained in the vector $\vec{V}$.

Therefore, both the summation based statistics and the comparison based statistics can be calculated in the proposed scheme.

#### Data Privacy

On the one hand, the adversary cannot infer the true position of the non-zero element from an encrypted vector, and thus can not recover *x*_*k*_ by using these information. There is a random function in the homomorphic encryption scheme. Even if two elements have the same value, their ciphertexts are still different from each other. For example, in the leaf nodes, each encoded vectors contain only a non-zero elements, and all other elements are zero. It’s easily infering the corresponding value *x*_*k*_ in plaintext domain by obtaining the position *i* of the non-zero elements and using $f_{m}^{-1}f_{e}^{-1}(i)$. However, in ciphertext domain, encrypted elements are different to each other, and even encrypted zero elements are also different to each other. As a result, inferring the position of non-zero elements is difficult in encrypted vectors.

**Algorithm 5** Operations on Server (Part III)

1: **procedure** obtain statistic result

2:  $CNT\leftarrow \sum_{i=1}^{L}n_{i}+\#mSet$                       ⊳ Count

2:  $\text{SUM}\leftarrow \sum_{i=1}^{L}f_{m}^{-1}(i)\times n_{i}+\sum_{mSet}x_{i}$               ⊳ Summation

4:  $\text{MEAN}\leftarrow \frac{SUM}{CNT}$                         ⊳ Average/mean

5:  *i*_*max*_ ← *max*({*i* *i* ∈ (0, *L*]&&*n*_*i*_ > 0})

6:  $max_{1}\leftarrow f_{m}^{-1}(i_{max})$

7:  *max*_2_ ← *max*(*mSet*)

8:  **MAX** ← *max* {*max*_1_, *max*_2_}                      ⊳ Maximum

9:  *i*_*min*_ ← *min*({*i* *i* ∈ (0, *L*]&&*n*_*i*_ > 0})

10:  $min_{1}\leftarrow f_{m}^{-1}(i_{min})$

11:  *min*_2_ ← *min*(*mSet*)

12:  **MIN** ← *min* *min*_1_, *min*_2_                        ⊳ Minimum

13:  *cnt*_*L*_ ← #{*mSet*(* < *MIN*_1_)},

                 ⊳ *cnt*_*L*_ is the total number in {*mSet*} whose value is less then or equal to *MIN*.

14:  $M\leftarrow min\left(\left\{j|\sum_{i=1}^{j}n_{i}\geq \left\lceil \frac{CNT}{2}\right\rceil -cnt_{L}\right\}\right)$

15:  $M^{\prime }\leftarrow min(\{j|\sum_{i=1}^{j}n_{i}\geq \left\lceil \frac{CNT+1}{2}\right\rceil -cnt_{L}\})$

16:  **if** CNT is odd **then**                          ⊳ Median

17:   $\text{MEDIAN}\leftarrow f_{m}^{-1}(M)$

18:  **else**

19:   $\text{MEDIAN}\leftarrow \frac{f_{m}^{-1}(M)+f_{m}^{-1}(M^{\prime })}{2}$

20:  **end if**

21:  $SUM({\hat{x}}^{2})\leftarrow \sum_{i=1}^{L}{\left(f_{m}^{-1}(i)\right)}^{2}n_{i}+\sum_{mSet}{x_{i}}^{2}$

22:  $E({\hat{x}}^{2})\leftarrow \frac{SUM({\hat{x}}^{2})}{CNT}$

23:  $E(\hat{x}{)}^{2}\leftarrow MEAN^{2}$

24:  **VAR** ← *E*(*x*^2^) − *E*(*x*)^2^                        ⊳ Variance

25:  $\text{STD}\leftarrow \sqrt{VAR}$                      ⊳ Standard deviation

26:  $m1cnt\leftarrow max(\vec{V})$

27:  $i_{mode1}\leftarrow find(\vec{V}==m1cnt)$

28:  *mode*2 ← *mode*(*mSet*)

29:  *m*2*cnt* ← *sum*(*mSet* = = *mode*2)

30:  **if** *m*1*cnt* > *m*2*cnt* **then**                        ⊳ Mode

31:   $\text{MODE}\leftarrow f_{m}^{-1}(i_{mode1})$

32:  **else**

33:   **MODE** ← *mode*2

34:  **end if**

35: **end procedure**

On the other hand, all message relayed or aggregated in the intermediate node are encrypted message. Due to the homomorphic encryption scheme, the aggregation for data in *R*_*d*_ performs on cipher text of ${\vec{v}}_{k}$ directly. Message in *aSet* and *bSet* are encrypted by a traditional encryption scheme. So all message relayed or aggregated in the intermediate node are encrypted message, all private keys keep in privacy only by the server. Without private key, no one can decrypt, so data confidentiality is achieved.

### A Concrete Example for SEDAR

Assuming *R*_*e*_ = (20, 40], *R*_*d*_ = (30, 34] and accuracy requirement is *a* = 1. As shown in Table 1, there are 10 nodes in the given network. Raw data of each node list in the 2nd column, the 3rd and 4th columns are data classification and processing results.

**Table 1:** Example for SEDAR.

ID	Raw data	Range	Result
1	32	*R*_*d*_	(0 1 0 0)
2	16	${\bar{R}}_{e}$	*aSet* = {“3”}
3	32	*R*_*d*_	(0 1 0 0)
4	33	*R*_*d*_	(0 0 1 0)
5	28	$R_{e}{\bar{R}}_{d}$	*mSet* = {28}
6	33	*R*_*d*_	(0 0 1 0)
7	34	*R*_*d*_	(0 0 0 1)
8	49	${\bar{R}}_{e}$	*aSet* = {“8”}
9	33	*R*_*d*_	(0 0 1 0)
10	25	$R_{e}{\bar{R}}_{d}$	*mSet* = {25}

Raw data of node 2 and node 8 are outside of the valid data range *R*_*e*_ = (20, 40]. Both of them will be regarded as illegal data and discarded, and their IDs will be added into alarm set *aSet*.

Raw data of node 5 and node 10 are in the boundary range $R_{b}=R_{e}{\bar{R}}_{d}=(20,30]⋃(34,40]$. Both of them will be added into boundary set *bSet*.

Other raw data are in the dominant range *R*_*d*_. Each of them will be transformed into *y*_*k*_ (*y*_*k*_ ∈ (0, 4]) by using the *mapping function*. Each valid mapped data *y*_*k*_ will then be encoded into a vector ${\vec{v}}_{k}$ whose length is L. The *y*_*k*_-th elements is 1, while all the remaining elements are set to 0. For example, in node 1, the raw data is *x*_1_ = 32, the mapped data *y*_*k*_ = 2 is obtained after mapping step, and in the encoding step, the 2nd (*y*_*k*_-th) element of the vector is set to 1, while other elements are 0, i.e. ${\vec{v}}_{k}=(0\;1\;0\;0)$.

Each vector will be encrypted by the homomorphic encryption scheme and in-network aggregation will perform directly in cipher domain.

Elements of *aSet* and *bSet* will be encrypted by the traditional encryption scheme, and be relayed to the server without in-network aggregation.

According the homomorphic property, the aggregation of vectors in cipher text domain is equivalent to that in plaintext. Therefore, the *server* can obtain the final aggregation result $\vec{V}=\sum {\vec{v}}_{k}$ by decrypting the received data. Encrypted data in *aSet* and *bSet* can also be decrypted by server. The final data obtained at the server include

$$\begin{array}{ccc} \vec{V} & = & \sum {\vec{v}}_{k}=\left(0\;2\;3\;1\right)\\ mSet & = & \cup mSet_{i}=\left\{25,28\right\}\\ aSet & = & \cup aSet_{i}=\left\{“3”,“8”\right\} \end{array}$$

Each statistic can be calculated using algorithm 5.

**CNT** = (2 + 3 + 1) + 2 = 8;

**SUM** = 2 × (2 + 30) + 3 × (3 + 30) + 1 × (4 + 30) + (25 + 28) = 250;

**MEAN** = 31.25;

*i*_*max*_ = 4, *max*_1_ = 34, *max*_2_ = 28, **MAX** = 34;

*i*_*min*_ = 2, *min*_1_ = 32, *min*_2_ = 25, **MIN** = 25;

*cnt*_*L*_ = 2, *M* = 2, *M*′ = 3, **MEDIAN** = 32.5;

*SUM*(*x*^2^) = 2 × (2 + 30)^2^+3 × (3 + 30)^2^+1 × (4 + 30)^2^+(25^2^+28^2^) = 7880; $E(x^{2})=\frac{SUM(x^{2})}{CNT}$; *E*(*x*)^2^ = *MEAN*^2^;

**VAR** = 8.4375; **STD** = 2.9;

*m*1*cnt* = 3; *i*_*mode*1_ = 3; *mode*2 = 25. (In *mSet*, 25 and 28 have the same frequency, and the first element, i.e., 25, is chosen as its mode.)

*m*2*cnt* = 1; because *m*1*cnt* > *m*2*cnt*, $\text{MODE}=f_{m}^{-1}(i_{mode1})=33$.

Note that CNT is 8 instead of 10; this is because there are two nodes whose data is out of the operation range, which means there a failure is caused by node failure or other reasons. That is to say, computation of the final statistics, can automatically adapt to the dynamic network.

## REDAR

In SEDAR, most elements of the encoded data $\vec{v}$ near the leaf nodes are zero, which means it contains redundant information. Directly transmitting these low information data using a full vector is too expensive. As these encrypted zeros are used to hidden the exact position of the encrypted non-zero elements. Encrypting all zeros is not necessary, especially when L large.

In this section, we propose REDAR, which can significantly reduce the communication cost with the trade-off of lower security on leaf node. In REDAR, all non-zero elements and a small number of random selected zero elements of the leaf nodes’ vector are encrypted.

### Random Encryption

Random selection zero-elements are used to reduce packet size, as well as provide security for the non-zero elements.

First, $\vec{v}$ are split the into several segments. Then, all non-zero elements and a small number of randomly chose zero elements are encrypted.

**Fig 3:**
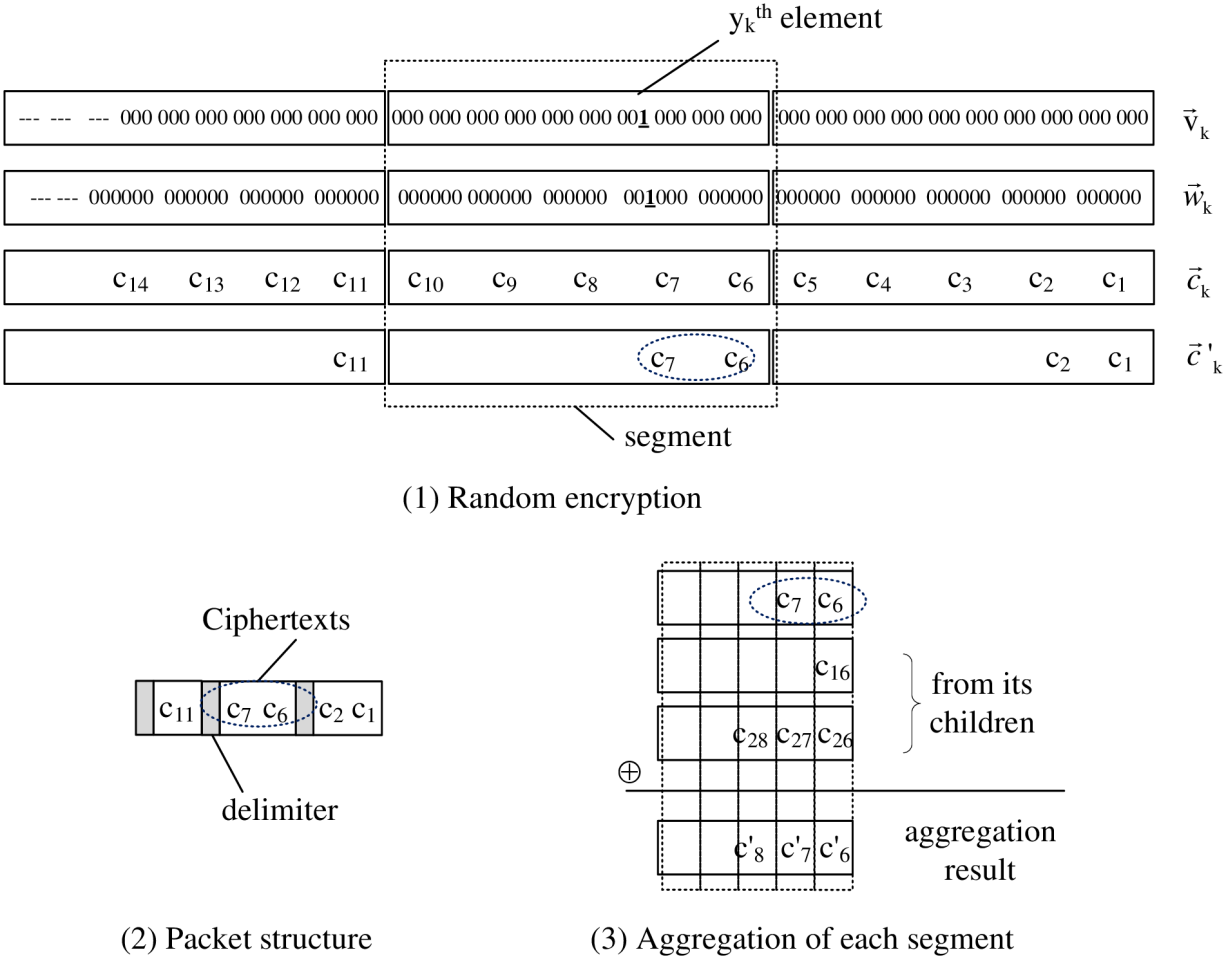
REDAR.

For example, in Fig 3-1, there are 27 elements in $\vec{v}$, each element contains 3 bits, each cipher element is encrypted from 2 elements, where the leftmost cipher element only contain 1 element in this case. $\vec{v}$ is split into 3 segments, each of the right two segments has 5 cipher elements at most, and the last segments has 4 cipher elements at most.

For each segment whose elements are zero, a random number *r* will generate between 1 and *n*_*s*_, where *n*_*s*_ is the number of elements in the segment. Then the *r* rightmost elements of the segment are encrypted using the homomorphic encryption scheme. For example, in Fig 3-1, all elements of the leftmost and the rightmost segments are zero, i.e., *n*_*s*_ = 4 and *n*_*s*_ = 5 respectively, and thus one cipher element in the leftmost segment and two cipher elements in the rightmost segment are obtained.

For the segment containing a non-zero element, a random number *r* is generated, where *r* is between 0 and *n*_*s*_ − *p*_*y*_, and *p*_*y*_ is the position of non-zero elements in the segment with respect to the right end. Then the *r* + *p*_*y*_ rightmost elements of the segment are encrypted. For example, in Fig 3-1, the 2*nd* segment contains a non-zero element, and *p*_*y*_ = 2. Because *r* = 0 is return by the random function, only *p*_*y*_ + *r* = 2 leftmost elements are encrypted.

### Packing and Unpacking

The packing step is used for constructing packet from the random encryption result. As show Fig 3-2, encrypted data in each segment are packed together in the original order, and a delimiter is added between adjacent segments. Because each cipher has the same size and only several leftmost elements are encrypted, the original encrypted vector can be reconstructed in the unpacking step.

### Secure Data Aggregation

All received packets are unpacked to get ${\vec{c}}_{k}^{\prime }$ sets, and then aligned together with the local generated encrypted data. Finally, data aggregation will performs directly on cipher domain column by column, and aggregation results of all segments are packed and send to its parent.

For example, in Fig 3-3, the first line is the encrypted data generated locally, the 2nd and 3rd are received from its children, and all of them are the 2nd segment of each vector. Segments received from different children may have a different number of elements, all of them are aligned to the right side. Because *c*_17_ is not exist in the 1st child, we ignore it, and just aggregate other two elements, i.e. ${c_{7}}^{\prime }=c_{7}⊕c_{27}$. Among these three segments, the maximum number of elements is 3 (the 3nd line in Fig 3-3), so the elements number in the aggregation result of this segment is also 3.

### Correctness and Security

As random selected encryption only performs on zero elements, all no-zero elements in each vector are encrypted, and aggregated into the final result. No raw data is loss in REDAR, so the final results are the same as that in SEDAR.

In REDAR, the communication cost is reduced for much less zero elements is encrypted and contained in the packet. However, as the number of encrypted data is reduced, it benefits the adversary with guessing the true position of non-zero elements. An inappropriate distribution of the encrypted data also benefits the adversary with the success probability of guess. So we need carefully design the random function and make sure that sufficient encrypted data is still retained after using random selected encryption. The more the encrypted elements, the lower probability it is guessed successfully. After aggregating at intermediate node, the encrypted elements will contain more than one encrypted non-zero elements, which means the success probability of the adversary will decrease significantly. More specifically, in the leaf node i, where only one non-zero elements in the vector. Assuming *n*_*i*_ encrypted data exist in the final packet, then the adversary’s success probability is $\frac{1}{n_{i}}$. In the cluster header, assuming k nodes aggregate together, the probability reduces to $\frac{1}{n^{k}}$, where *n* = ∑_*j*_ *max*_*i*_(*n*_*ij*_), and *n*_*ij*_ is the number of elements in the jth segment of node i. As n and k increase along the aggregation tree, the adversary’s success probability decrease obviously.

For example, when *n* ≥ 25 and *k* ≥ 4, the success probability no larger than 2.56 × 10^−6^. As in cluster-based networks, the cluster member in each cluster is often large than 4, which means when *n* ≥ 25, except in the leaf node, no encrypted data can be success guessed with probability larger than 2.56 × 10^−6^. When k = 6 and n = 35, the success probability already decreases to 5.44 × 10^−10^.

## CEDAR

In this section, we present CEDAR. CEDAR and REDAR are complementary schemes. CEDAR is used before *encoding*, while REDAR is used after *encoding*.

**Fig 4:**
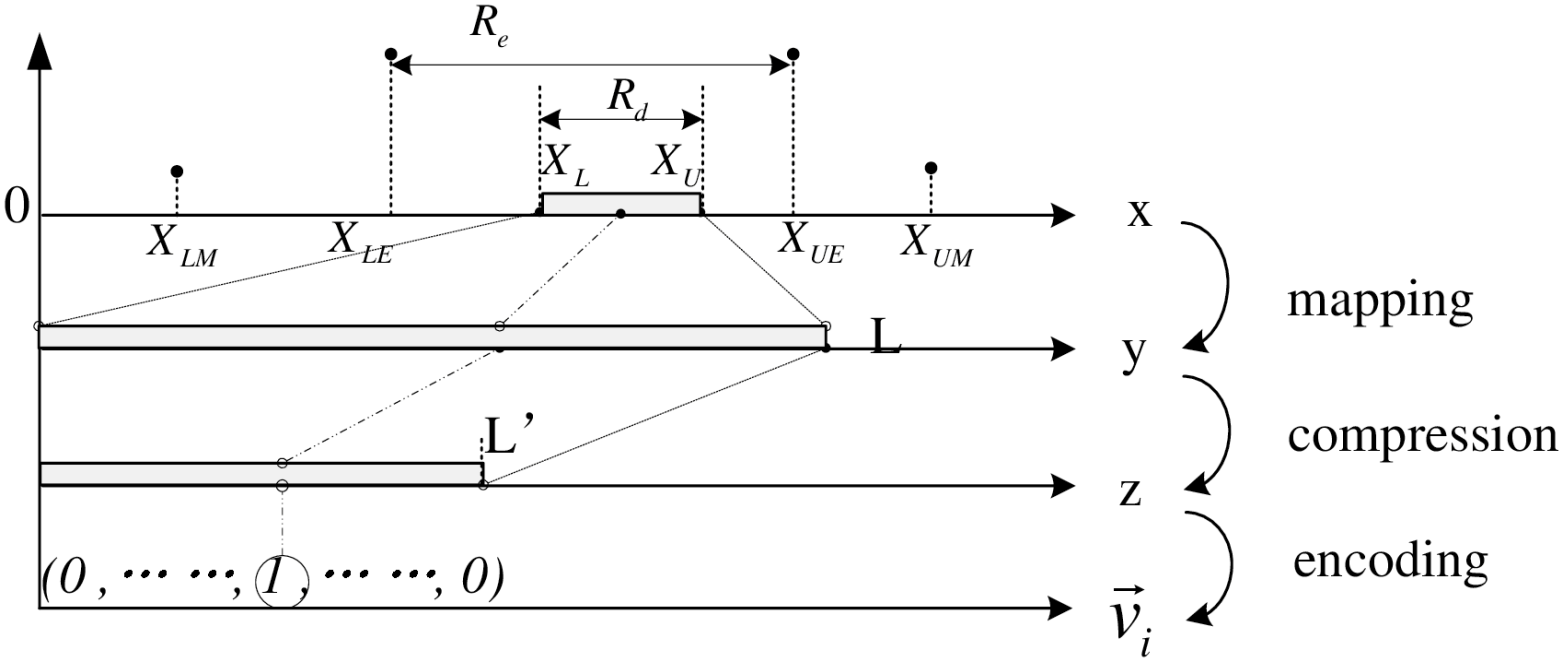
CEDAR.

In SEDAR, the total communication cost is mainly determined by the size and accuracy of *R*_*d*_, i.e. $L=\frac{\left|R_{d}\right|}{a}$. L sometimes is large, so the total communication cost is still heavy. In order to reduce the communication cost, a *compression* step is introduced in CEDAR. As shown in Fig 4, mapping data *y* is compressed from a lager space with size of *L* into a smaller space with size of *L*′. Encoding step executes on compression data *z*, which make the vector length decreased from *L* to *L*′. Compression function can be a linear one or a non-linear one. Due to the limited space, we only illustrate the linear one.

A linear *compression* function *f*_*c*_ can compress *y* into *z*, i.e., $z=f_{c}(y)=f_{c}f_{m}(x)=\left\lceil \frac{y}{c}\right\rceil \;=\;\left\lceil \frac{f_{m}(x)}{c}\right\rceil$, *c* is the compression factor, which is larger than 1. Encoding step is based on *z* instead of *y*, i.e., $\vec{v}=f_{e}(z)=f_{e}f_{c}(y)$. So the total communication cost is reduce from *L* to $\left\lceil \frac{L}{c}\right\rceil$. One can recover $\hat{y}$ as an estimate of *y*, using the decompressing function on *z*, i.e., $\hat{y}=f_{c}^{-1}(z)=f_{c}^{-1}f_{e}^{-1}(i)\;=\;c\;\times \;z\;-\;\left\lfloor \frac{c}{2}\right\rfloor \;=\;c\;\times \;i\;-\;\left\lfloor \frac{c}{2}\right\rfloor$.

## Performance Analysis

In this section, we analyse communication and computation performance of proposed schemes. Performance criteria includes whether there are existing a bottleneck, and whether it achieves load balance.

### Communication performance

First, we analyse the maximum packet size to judge whether exists a bottleneck. Then analyse the distribution attribution of packet size to judge whether it achieves load balance. After a thorough analysis, we find out that no bottleneck exist, and it achieves load balance. The detailed analysis goes as follows.

Data in *R*_*d*_ are encrypted by a homomorphic scheme, so encrypted data can aggregate directly in cipher domain in the intermediate nodes, and thus the total length will not change. Data in *R*_*b*_ uses a traditional encryption scheme. Without the private key, the intermediate nodes have to cascade each encrypted data and replay forwarding. So the length will increase, the minimum values of the data packets size appear in the leaf node of the aggregation tree, and the maximum length of the package is in the vicinity of the server node.

Now, let’s analyse packet length for *R*_*d*_ and *R*_*b*_ respectively. For the sake of simplicity, data length analysis is based on plain text.

Communication cost for *R*_*d*_ is determined by the number of elements in the vector and the data length of each element. The former is determined by the range length of *R*_*d*_ and accuracy requirement *a*. The latter determined by the largest number of samples fall in the same point, and the worst case is all samples in *R*_*d*_ (i.e. *N* × *P*(*R*_*d*_)) fall in the same position. In practice, the probability of the worst case can be ignored. So, $Cost_{R_{d}}<\frac{\left|R_{d}\right|}{a}\lceil log(N\times P(R_{d}))\rceil =\frac{2\beta \hat{\delta }}{a}\lceil log(N\cdot P(R_{d}))\rceil$.

Now, let’s consider the communication cost for *R*_*b*_. Since the data in *R*_*b*_ is not aggregated in the intermediate nodes, the total data length will reach the maximum in the vicinity of the server node. The maximum value is determined by the total number of samples *N* and the probability (*P*(*R*_*b*_)) of the sample in the region *R*_*b*_, as well as the transmission overhead of a single sample. In practice, we can assume that the probability of abnormal data is much less than the normal one, which means the number of elements outside *R*_*e*_ can be ignored. So, $P(R_{b})=P(R_{e}\bar{R_{d}})=P(R_{e})-P(R_{d})\approx 1-P(R_{d})$. and then $Cost_{R}{}_{{}_{b}}\;<\text{ N}\times \text{P}({\text{R}}_{b})\left\lceil \text{log}\frac{R_{b}}{a}\right\rceil \;\approx \text{ N}(1-P({\text{R}}_{d}))\;\left\lceil \text{log}\frac{\left|{\text{R}}_{b}\right|\;-\;\left|{\text{R}}_{d}\right|}{a}\right\rceil$.

In order to judge wether it achieves load balance, let’s analyze the distribution of the whole network traffic first.

The minimum values of the data packets size appear in the leaf node of the aggregation tree. In the leaf node, when *x* ∈ *R*_*b*_, no encoding step is used, so its data length is $\left\lceil log\frac{\left|R_{e}|-|R_{d}\right|}{a}\right\rceil$. When *x* ∈ *R*_*d*_, the encoding data length is $Cost_{R_{d}}$. The former one (Let’s denote it as *C*_0_) is much less than the last one. However, *C*_0_ only exists in very small number of leaf node. Assuming each cluster contains *m* leafs. The probability that *C*_0_ appears at the same time in *m* nodes is (1 − *P*(*R*_*d*_))^*m*^. For example, in a normal distribution, assuming *β* = 2, when m = 3, (1 − *P*(*R*_*d*_))^*m*^ = 9.48 × 10^−5^. The probability is small, which means even a small number of leaf node has a packet size of *C*_0_, its parent will at least $Cost_{R_{d}}$. Therefore, *C*_0_ is lack of significance, and the representative minimum value should be selected as $Cost_{min}=Cost_{R_{d}}$.

In the whole network, the minimum packet appears in the leaf nodes, the maximum packet appears in the vicinity of the server. In the path of the leaf node to the root node, the size of the packet increases from the minimum to the maximum value. Because *P*(*R*_*d*_) ≫ *P*(*R*_*b*_), the growth rate of packet size is small enough, and the average packet size $\bar{Cost}\approx Cost_{R_{d}}$.

The maximum value of the data packet is, $Cost_{\text{max}}=Cost_{R}{}_{{}_{d}}+Cost_{R}{}_{{}_{d}}$. On the one hand, once the *R*_*d*_ is determined, $Cost_{R_{d}}$ can be treat as a const. If $Cost_{R_{d}}$ is too large, CEDAR can be used. So $Cost_{R_{d}}$ is controllable. On the other hand, according the define of *R*_*d*_, *P*(*R*_*d*_) ≈ 1, so $Cost_{R_{d}}$ is small enough. As a result, the maximum packet size can be regarded as a controllable const, and there is no bottleneck in the network.

As the difference among *Cost*_*min*_, *Cost*_*max*_ and $\bar{Cost}$ is small, we can easily make a conclusion that it achieves load balance.

### Computation performance

For computation performance, we also analyse the maximum computation cost to judge whether exists a bottleneck, and analyse the distribution attribution of computation cost to judge whether it achieves load balance. We find out that no bottleneck exist, and it achieves load balance. The detailed analysis goes as follows.

Each data can either be homomorphic encrypted after encoded, or encrypted by traditional scheme directly, according the range it belongs to. Let’s denote the computation cost of the former as *C*_11_, and the latter one as *C*_21_.

For the data inside the *R*_*d*_, mapping and encoding are required before homomorphic encryption. Both of them cost much less than encryption and decryption, thus can be ignored. For the homomorphic encrypted data, the intermediate nodes will not decrypt it, and aggregate them directly in cipher domain. Assuming a single cipher domain addition cost *C*_13_, the total aggregation cost is *C*_13_(*N* × *P*(*R*_*d*_) − 1), due to that *N* × *P*(*R*_*d*_) − 1 times aggregation operation are necessary for *N* × *P*(*R*_*d*_) elements in *R*_*d*_.

The final aggregated result will be decrypted in the server, and the decryption cost is *C*_12_. Each encrypted data in the boundary range, will also decrypted in the server, and the decryption cost is *C*_22_.

So the total computational cost is *C* = *C*_11_ *N* × *P*(*R*_*d*_) + *C*_13_(*N* × *P*(*R*_*d*_) − 1)+*C*_12_ + *N* × *P*(*R*_*b*_)(*C*_21_+*C*_22_).

Average computational cost is $C_{a}{}_{vg}\;=\;\frac{C}{N}\;=\;C_{1}{}_{1}P(R_{d})+\frac{C_{1}{}_{3}(N\times P(R_{d})-1)}{N}+\frac{C_{1}{}_{2}}{N}+P(R_{b})\;(C_{21}+C_{22})\;\approx \;(C_{11}+C_{13})\;P(R_{d})+(C_{21}+C_{22})\;(1-P(R_{d}))$.

In instances of homomorphic encryption, encryption cost and decryption cost are often much larger than the cipher domain aggregation cost. For example, in the ECC-based version, the main operations of encryption and decryption are scalar multiplication, and the main operation of cipher domain aggregation is point addition. The former is far greater than the latter, so *C*_13_ can also be ignored, and *C*_*avg*_ ≈ *C*_11_ *P*(*R*_*d*_) + (*C*_21_+*C*_22_)(1 − *P*(*R*_*d*_)).

There are two main computational cost operations, i.e. encryption and decryption. Both of them not exist in the same node. Each client only performs one type of encryption operation, i.e. homomorphic one or traditional one. Two types of decryption exist in the server.

Since each client only chooses one of the two kinds of encryption mechanisms, each data is encrypted only once, so the computation cost is *C*_11_ or *C*_21_. In general, the encode data has larger length than the raw data, so *C*_11_ > *C*_21_, so the maximum computational cost of client is *C*_11_. Two types of decryption exist in the server, the corresponding overhead is *C*_12_ + *N* × *P*(*R*_*b*_)*C*_22_. Because *N* × *P*(*R*_*b*_) is often small, and the server node has a large computational power, the decryption operation is not a difficult task. Therefore, there is no computational bottleneck exist.

Each client only encryption once, and the aggregation operations in each intermediate node are not compute-intensive, so the the proposed scheme also achieve load balance in computation.

### Statistics functions supported

In this section, we compare the proposed schemes with other data aggregation schemes on statistics functions and encoding method. Table 2 is the comparison result. All of them are distributed aggregation schemes, which mean that intermediate nodes generate partial aggregation results from their received data.

**Table 2:** Comparison on Statistics Functions and Encoding Method.

	Encoding	Statistics
Considine et al. [9]	Synopsis generation function	CNT, SUM, AVG VAR, STD
Roy et al. [11]		
Li et al. [17]	Slicing and assembling technique	
Yang et al. [18]		
Castelluccia et al. [10]	No	
Lu et al. [19]		
Ertaul et al. [20]	No	MAX, MIN
Samanthula et al. [21]		
RCDA [14]	*l* = ⌈*logL*⌉, *β* = *l*(*i* − 1), ${\vec{v}}_{i}=x_{i}||0^{\beta }$	CNT, SUM, AVG VAR, STD, MAX MIN, MODE, MEDIAN
EERCDA [13]		
Proposed schemes	${\vec{v}}_{i}=zeros(1,L)$,${\vec{v}}_{i}(y_{i})=1$	

## Evaluation

### Data sets description

Evaluation is based on six datasets gathered from different type of sensors. All of them are obtained from TAO (Tropical Atmosphere Ocean) project. The TAO is a project of NOAA (National Oceanic and Atmospheric Administration), which aim to enable real-time collection of high quality oceanographic and surface meteorological data for monitoring, forecasting, and understanding of climate swings associated with El Nino and La Nina.

Table 3 is the general description of each dataset. *Rh*0*n*156*e*_*hr* is a dataset of relative humidity. *Bp*0*n*156*e*_*hr* is sea level pressure. *W*0*n*156*e*_*hr* is wind direction. *Sst*0*n*147*e*_*hr* and *sst*0*n*156*e*_*hr* are different datasets of sea surface temperature. *rad*0*n*156*e*_*hr* is shortwave radiation. The 2nd column is the sample size of each dataset. The 3rd and 4th columns are skewness and kurtosis respectively. The 5th and 6th columns are mean and standard deviation estimated using the history record.

**Table 3:** General description of datasets.

Dataset	Size	Skewness	Kurtosis	$\hat{\delta }$	$\hat{\mu }$
rh0n156e_hr	144250	0.331	3.301	5.508	78.587
bp0n156e_hr	122035	-0.174	2.852	1.820	1008.3
w0n156e_hr	146702	-0.409	1.992	96.268	197.416
sst0n147e_hr	65535	0.313	3.706	0.469	29.712
rad0n156e_hr	39916	-0.008	1.757	234.273	596.817
sst0n156e_hr	4672	0.829	4.683	0.358	29.338

These datasets cover several different scenarios. The distribution characteristics of them are different from each other, and thus has certain representativeness.

### Effectiveness of Range Segmentation Model

In the proposed schemes, a range segmentation model is introduced to reduce the encoded vectors length, and thus reduce the total communication cost as long as the *P*(*R*_*b*_) is small enough. To achieve this purpose, we should choose dominate range carefully and make sure that samples beside this range is small enough. Now, let’s verify whether the boundary setting of the dominate range (*R*_*d*_) is effective.

As we described above, the lower bound *X*_*L*_ and the upper bound *X*_*U*_ of dominate range *R*_*d*_ are determined by $X_{L}=max(X_{LE},\hat{\mu }-\beta \hat{\delta })$ and $X_{U}=min(X_{UE},\hat{\mu }+\beta \hat{\delta })$. *X*_*LE*_ and *X*_*UE*_ are const defined in TAO project. $\hat{\mu }$ and $\hat{\delta }$ are estimated from history data, which can also be regarded as const. Different dominate range can be generated by different *β*.

The proportion of data outside *R*_*d*_, i.e. *P*(*R*_*b*_), under different parameters are list in Table 4. As *β* increase, *P*(*R*_*b*_) reduce significantly. For example, when *β* = 2.2, *P*(*R*_*b*_) is no larger than 3.5% in all six datasets, and two of them are even reduced to zero.

**Table 4:** *P*(*R*_*b*_) in different dominant range setting.

Dataset	*β* = 1.4	*β* = 1.8	*β* = 2.2	*β* = 2.6	*β* = 3
rh0n156e_hr	15.61%	7.39%	3.22%	1.40%	0.50%
bp0n156e_hr	16.40%	6.78%	2.54%	0.66%	0.19%
w0n156e_hr	17.11%	4.82%	0	0	0
sst0n147e_hr	14.10%	6.73%	3.27%	1.55%	0.75%
rad0n156e_hr	18.70%	0	0	0	0
Sst0n156e_hr	14.75%	5.78%	3.47%	1.85%	1.02%

However, it’s not means the larger of *β*, the better of the communication performance. As *β* increase, the encoded vector length also increase, and the communication cost for *R*_*d*_ will increase. We need to achieve a balance between the communication cost for *R*_*d*_ and *R*_*b*_.

**Fig 5:**
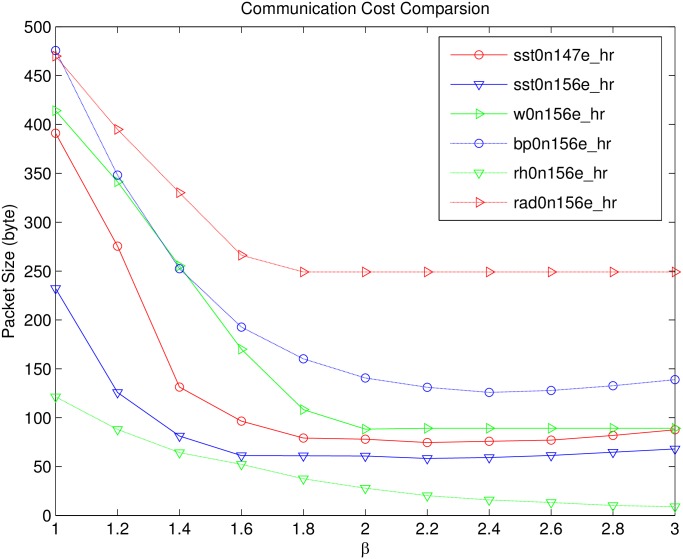
Communication cost in different *R*_*d*_ setting.

Fig 5 is the relationship between the maximum packet size and dominant range *R*_*d*_ setting. The network size is 1000. We can easily find out that, as *β* increasing, the maximum packet size reduces significantly, and when *β* > 1.8, the decreasing rate becomes moderate. Let’s analysis the reason. As the increase of *β*, the communication cost for the boundary range *R*_*b*_ reduce significantly. More specifically, the communication overhead reduced in *R*_*b*_ is much larger than that increased in *R*_*d*_, so the total packet size is still significantly decreased. When *β* reaches a certain value, the maximum packet size reaches a minimum value. E.g., *β* = 1.6 for *sst*0*n*156*e*_*hr* and *β* = 2 for *w0n156e*. In some case, when *β* is larger than its optimal value, *P*(*R*_*b*_) is small enough, the communication cost for *R*_*b*_ can be ignored, and the cost for *R*_*d*_ is may increase as the bound of *R*_*d*_ still in *R*_*e*_, so the maximum packet size may increase mildly.

### Communication Cost of SEDAR in Different *R*_*d*_ Setting

Fig 5 is the communication cost of SEDAR in different *R*_*d*_ setting. According to this figure, when *β* is between 1.8 and 3, the change of the maximum packet size in each dataset is relatively small. Which means, any *β* ∈ [1.8, 3] meets the basically requirements and doesn’t significantly reduce the communication performance. This feature is very useful, which means *R*_*d*_ setting is easy.

Although it is difficult to achieve optimal performance by setting an accurate dominant range in advance, by choosing arbitrary *β* ∈ [1.8, 3], we can still obtain a suboptimal performance, which is very similar to the optimal one. For example, in following evaluation, we directly set *β* = 2 for different datasets, and still obtain a good result.

### Comparsion with RCDA and EERCDA

**Fig 6:**
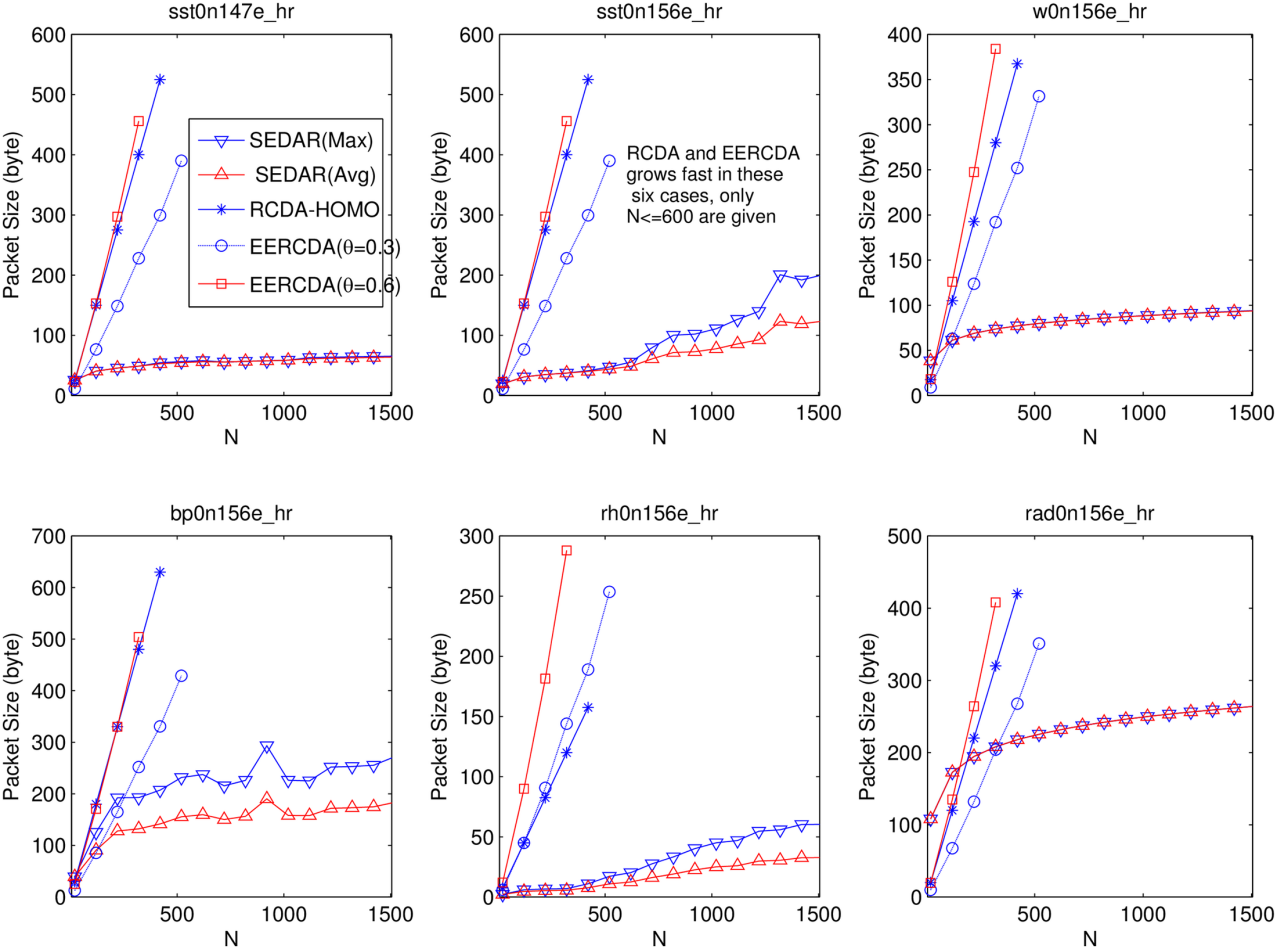
Comparsion with RCDA and EERCDA.

In this section, we compare SEDAR with RCDA and EERCDA. Both of them support mutil-functional security data aggregation, and homomorphic encryption scheme is used in all of them. The main difference lies in the encoding function. Each client encrypts collected and encoded data. The aggregation performs on cipher text directly at each intermediate aggregator, and the decryption performs on the server. The computation cost of encryption and decryption are near-linear related to the encoded data length. Limited to space, we only concern the comparison on communication cost. In these evaluations, *β* = 2. The comparison result lists in Fig 6. (*θ* is used to characterize the intensity of data fluctuation in a given application for EERCDA.)

According Fig 6, we can easily find out that the proposed scheme is obviously superior to RCDA and EERCDA. And due to the slow growth of communication cost as the increase of N, it can be applied to large scale networks.

In *w*0*n*156*e*_*hr*, *bp*0*n*156*e*_*hr* and *rad*0*n*156*e*_*hr*, when N is small, RCDA and EERCDA is better than SEDAR. In these datasets, when *β* = 2, the dominate range is a little large. So when the network size N is small, the communication cost is larger than that in RCDA and EERCDA. However, when N increases to a certain value, the advantage of this scheme is very obvious. In other three datasets, the dominate region size is small, and most samples are in the dominate range when *β* = 2, so the proposed scheme has an absolute advantage even in the small network.

In *sst*0*n*147*e*_*hr*, *w*0*n*156*e*_*hr* and *rad*0*n*156*e*_*hr*, the average and maximum communication cost are almost have the same value, this is because most elements are in *R*_*d*_. In contrast, the average and maximum communication cost aren’t the same in other three datasets.

Table 5 is comparison on end-to-end aggregation time. Due to limited space, we only compare SEDAR with RCDA. The evaluation is built on MICAz. MICAz has a low-power 8-bit microcontroller ATmega128L and an IEEE 802.15.4 compliant CC2420 transceiver. The clock frequency of ATmega128L is 8 MHz. The claimed data rate of CC2420 is 250 kbps. Meulenaer et al. [22] measured the effective data rate for transmitting is 121 kbps which far below the claimed rates. In the energy models used in this paper, we use 121 kbps as the data rate for the evaluation of communication delay. For the computational cost evaluation, we decide to implement the proposed scheme based on TinyECC [23]. According to its evaluation result based on MICAz, the execution time for encryption is 3907.46ms, which is similar to the one used in RCDA [14]. According to RCDA, MICAz needs 73.71 ms to aggregate two data in cipher domain. According to the comparison results, end-to-end aggregation time of SEDAR is much smaller than that of RCDA. With the increase of network size, this advantage will be more obvious.

**Table 5:** Comparison on end-to-end aggregation time (unit: ms; N = 1000; cluster-based network. Compu.: computation delay; Commu.: communication delay; Total: total delay).

	SEDAR			RCDA		
	Compu.	Commu.	Total	Compu.	Commu.	Total
sst0n147e_hr	1.52 × 10^4^	15.59	1.52 × 10^4^	4.96 × 10^5^	339.25	4.96 × 10^5^
sst0n156e_hr	1.15 × 10^4^	20.63	1.15 × 10^4^	4.35 × 10^5^	339.25	4.35 × 10^5^
w0n156e_hr	2.30 × 10^4^	23.54	2.30 × 10^4^	4.37 × 10^5^	237.48	4.37 × 10^5^
bp0n156e_hr	2.33 × 10^4^	42.06	2.34 × 10^4^	7.56 × 10^5^	407.11	7.56 × 10^5^
rh0n156e_hr	1.29 × 10^3^	6.65	1.29 × 10^3^	7.97 × 10^4^	101.77	7.98 × 10^4^
rad0n156e_hr	6.50 × 10^4^	66.48	6.51 × 10^4^	1.05 × 10^6^	271.40	1.05 × 10^6^

### Cost and Accuracy Evaluation for CEDAR

**Fig 7:**
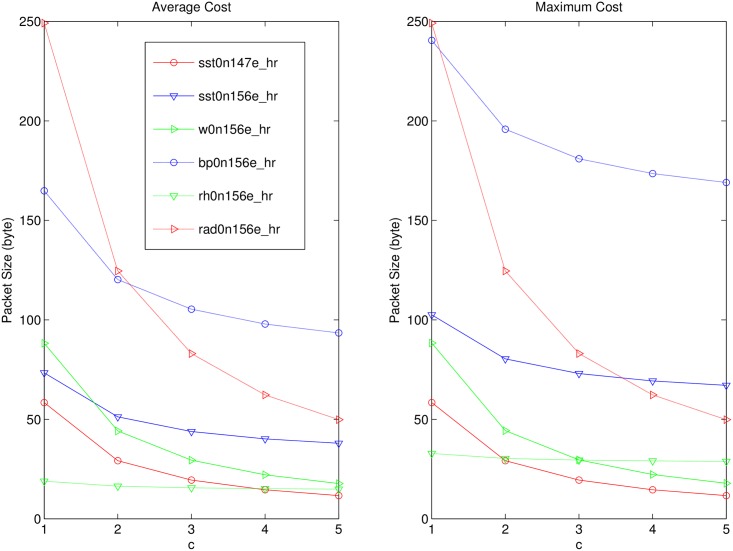
Cost Evaluation of CEDAR.

**Fig 8:**
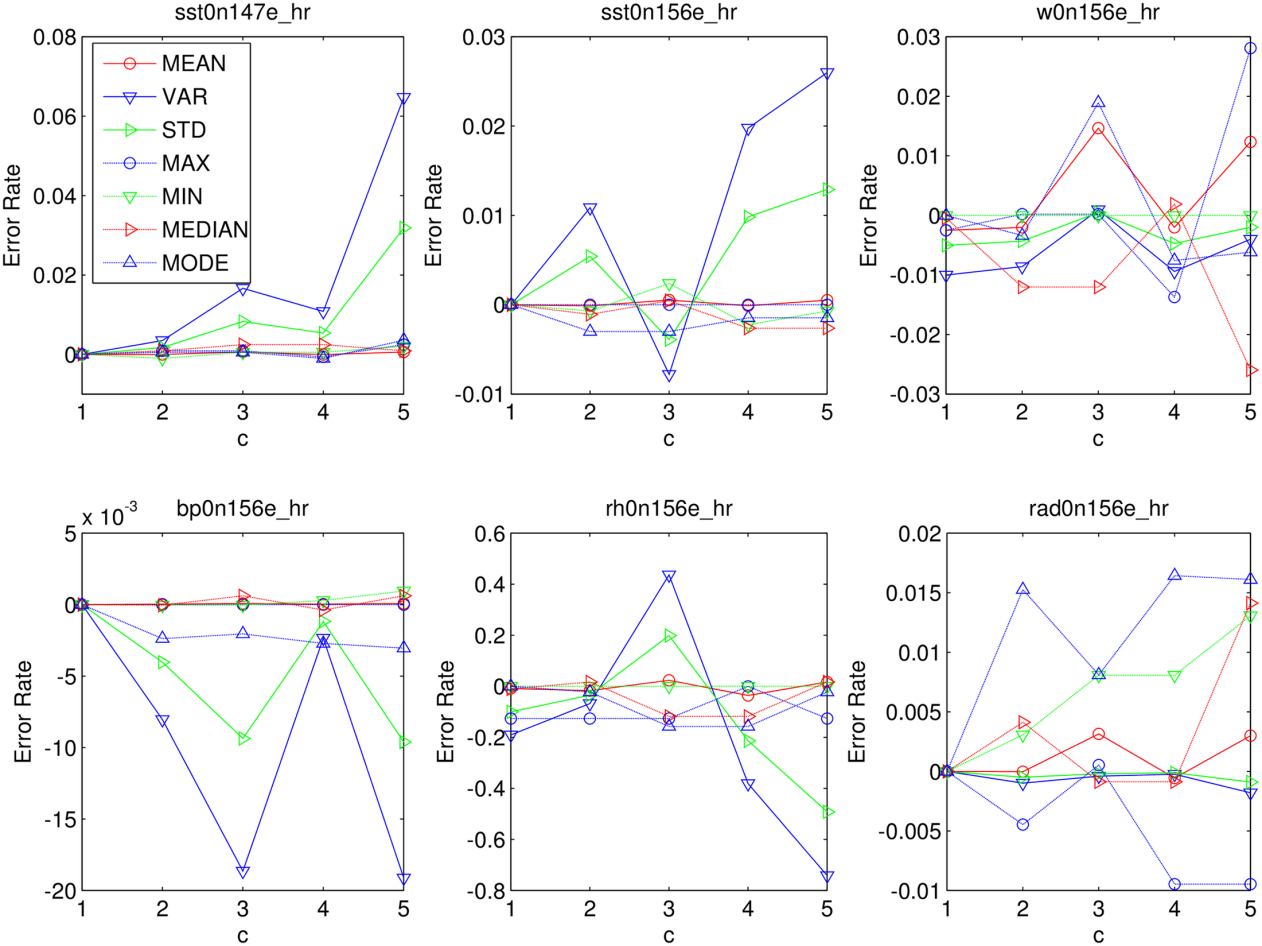
Accuracy Evaluation of CEDAR.

We measure the performance of CEDAR in this section. Figs 7 and 8 are the communication overhead and accuracy in different compression factor *c*.

According to Fig 7, we can know that with the increase of *c*, the average and the maximum communication cost of each dataset are reduced to some extent. The reduction trend in each dataset is not entirely consistent. When the compression factor is large, the communication volume curve is flat, which means the compression effect is decreased. According to Fig 8, with the increase of C, the error rates in each dataset are increasing. So it is necessary to ensure that a balanced between the communication cost and the error rate.

The growth trend of the error rate has a certain degree of relationship with the initial communication cost. The initial communication cost is the corresponding communication cost in SEDAR. The error rates increase more quickly in cases that have much larger initial communication cost. For example, as shown in Fig 8, the initial communication cost of *rad*0*n*156*e*_*hr* is relatively large, and when *c* = 5, the bound of error rates is still less than ±0.015. In *sst*0*n*147*e*_*hr*, *w*0*n*156*e*_*hr*, and *rh*0*n*156*e*_*hr*, in which the initial communication cost is small, the bound is close to or more than *pm*0.03 when *c* = 5. In particular, error bound of *rh*0*n*156*e*_*hr*, is greater than 0.4 when *c* = 4. In fact, according to Fig 7, the initial communication cost of *rh*0*n*156*e*_*hr* is the minimal one, and the reduction tendency of *rh*0*n*156*e*_*hr* is not obvious.

Hence, we can choose a large compression factor for the case with large initial communication cost, and we should choose a small one, or even give up the CEDAR for the case with small initial communication cost.

## Related work

### Distributed Aggregation

Distributed aggregation is a traditional research topic in database community. Kuhn and Oshman [6] studied the complexity of computing count and minimum in synchronous directed networks. Hobbs et al. [7] presented a distributed protocol to compute maximum and average under the SINR model. Cormode and Yi [8] focused on tracking the value of a aggregation function on distributed monitoring area. Cheng et al. [24], Li and Cheng [25] considered the approximate aggregation problem and presented (*ϵ*, *δ*)-approximate schemes based on Bernoulli sampling. Xie and Wang [26] and Shen et al. [27] studied network construction and message routing algorithm for data aggregation.

### Secure Distributed Aggregation

Several secure distributed aggregation schemes have been proposed. Most of them focus on secure itself, and very limited numbers of aggregation functions can be supported. Considine et al. [9] and Roy et al [11] proposed secure distributed aggregation scheme for duplicate sensitive aggregation based on synopsis generation function. Li et al. [17] and Yang et al. [18] proposed slice-mix based schemes for additive aggregation functions, which guarantees data privacy through data “slicing and assembling” technique. Castelluccia et al. [10] and Lu et al. [19] proposed secure distributed aggregation scheme based on homomorphic encryption, which is also only support summation-based statistical functions, such as CNT and SUM. Agrawal et al. [28] presented the order-preserving encryption scheme. Ertaul et al. [20] and Samanthula et al. [21] applied it to secure distributed aggregation, to get comparison-based statistics, such as MAX, MIN. However, summation-based statistics is not support in these schemes. Chien-Ming et al. [14] and Jose et al. [13] adopted encoding steps before encryption to achieve arbitrary aggregation function. However, their encoding steps are simple concatenation all sensing data without any information compression method, and the communication cost is too heavy to extend to large scale networks. Enabling operation in cipher domain is also an important topic in cloud computing [29–31]. In addition to traditional encryption scheme, data privacy can be achieved by steganography [32, 33]. Beside data privacy, date authentication is also necessary. Ren et al. [34] proposed an efficient mutual verifiable provable data possession scheme. Guo et al. [35] designed a lightweight and tolerant authentication to guarantee data security.

## Conclusions

In this paper, we have studied the problem of *multifunction secure distributed aggregation*, and also have proposed three complementary schemes (i.e., SEDAR, REDAR and CEDAR) to solve this problem. The first one can obtain accurate aggregation results. The other two can significantly reduce communication cost with the trade-off lower security and lower accuracy, respectively. Extensive analysis and experiments, based on six different scenes of real data, have shown that all of them have an excellent performance.
